# Pediatrics Consequences of Caesarean Section—A Systematic Review and Meta-Analysis

**DOI:** 10.3390/ijerph17218031

**Published:** 2020-10-31

**Authors:** Aneta Słabuszewska-Jóźwiak, Jacek Krzysztof Szymański, Michał Ciebiera, Beata Sarecka-Hujar, Grzegorz Jakiel

**Affiliations:** 1 First Department of Obstetrics and Gynaecology, Centre of Postgraduate Medical Education, Żelazna 90 Street, 01-004 Warsaw, Poland; jkszymanski2@gmail.com (J.K.S.); grzegorz.jakiel1@o2.pl (G.J.); 2 Second Department of Obstetrics and Gynaecology, Centre of Postgraduate Medical Education, Cegłowska 80 Street, 01-809 Warsaw, Poland; michal.ciebiera@gmail.com; 3 Department of Basic Biomedical Science, Faculty of Pharmaceutical Sciences in Sosnowiec, Medical University of Silesia in Katowice, Kasztanowa 3 Street, 41-200 Sosnowiec, Poland; beatasarecka@poczta.onet.pl

**Keywords:** caesarean section, neonatal outcomes, respiratory disorders, asthma, obesity, overweight, neurological disorders

## Abstract

Background: Cesarean section is a surgical procedure, which is the most frequently performed in gynecology and obstetrics. It is commonly believed that an operative delivery is a less painful and safer mode of delivery, which translates into an increasing number of the procedures performed without medical indications. The maternal sequelae of cesarean sections are well elucidated and widely discussed in the literature, while long-term neonatal consequences still remain the issue of research and scientific dispute. The aim of the present paper was to perform a systematic review of current literature regarding pediatrics consequences of cesarean section. Methods: We reviewed available data from PubMed, Science Direct as well as Google Scholar bases concerning early and long-term neonatal sequelae of operative deliveries. The following key words were used: “cesarean section”, “caesarean section”, “neonatal outcomes”, “respiratory disorders”, “asthma”, “obesity”, “overweight”, and “neurological disorders”. A total of 1636 papers were retrieved out of which 27 were selected for the final systematic review whereas 16 articles provided data for meta-analysis. Statistical analyses were performed using RevMan 5.4. To determine the strength of association between the caesarean section and respiratory tract infections, asthma, diabetes type 1 as well as obesity the pooled odds ratios (OR) with the 95% confidence intervals (CI) were calculated. Results: Conducted meta-analyses revealed that caesarean section is a risk factor for respiratory tract infections (pooled OR = 1.30 95%CI 1.06–1.60, *p* = 0.001), asthma (pooled OR = 1.23 95%CI 1.14–1.33, *p* < 0.00001) as well as obesity (pooled OR = 1.35 95%CI 1.29–1.41, *p* < 0.00001) in offspring. Conclusions: The results of the studies included indicated that children delivered by cesarean section more commonly developed respiratory tract infections, obesity and the manifestations of asthma than children delivered vaginally. The risk of developing diabetes mellitus type 1 or neurological disorders in offspring after caesarean section is still under discussion.

## 1. Introduction

Cesarean section is the most common surgical procedure performed in women worldwide. Notably, a high percentage of surgical deliveries did not translate into reduced maternal or neonatal mortality [1,2,3,4,5]. According to the recommendations of the World Health Organization (WHO) the rates of cesarean sections should range between 10 and 15% [6]. However, the fear of labor pains and simultaneous concern about the baby to be born, are more and more commonly leading to women choosing to deliver by cesarean section [7,8]. The probability of complications secondary to the implemented procedure increases with the increasing percentage of cesarean sections. Globally, perinatal mortality rates reach 19 out of 1000 children [9]. As regards African countries—1 in 23 neonates dies as a consequence of cesarean section [10], while in developed countries it is believed that the procedure may prevent severe perinatal complications.

According to an increasing number of epidemiologic studies, children delivered by cesarean section more commonly developed respiratory and neurological disorders (e.g., autism spectrum disorders [11], schizophrenia [12]) and immune-related diseases, such as asthma [13,14], skin atopy [15], juvenile arthritis, coeliac disease [16], type 1 diabetes (T1D) [17] or obesity [1,18,19,20,21,22,23]. It is worth noting differences between the occurrence of the above-mentioned conditions in cases when the surgical procedure was performed after delivery had started.

Hypothetical Mechanisms of the Influence of Cesarean Section on Neonatal Status

**Figure 1:**
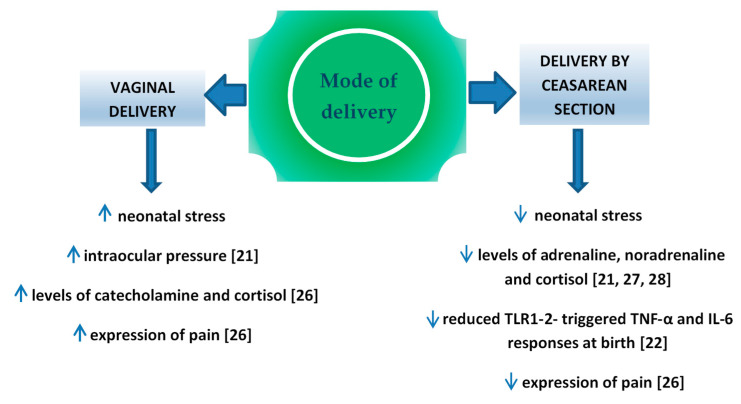
Factors influence on fetus, depend on mode of delivery.

Perinatal stress leads to the increased production of catecholamine and cortisol in the infant’s blood [21]. It is important for the development of pulmonary maturity [24] and the adaptation of the circulatory system to extrauterine life [25]. The completion of pregnancy without associated neonatal stress (i.e., in case of an elective cesarean section) is a potential factor which may interfere in those processes. Schuller et al. claimed that neonates delivered vaginally were characterized by higher cortisol levels and presented higher expression of pain compared to children delivered by cesarean section [26]. In the case of infants delivered by cesarean section, the level of cortisol measured in the umbilical cord blood was significantly lower compared to vaginally delivered neonates [27,28,29] or to vacuum-assisted delivery [30], which might result in the increased percentage of adaptation complications, such as respiratory distress syndrome (RDS), persistent tachypnea or pulmonary hypertension which require hospitalization in the neonatal intensive care unit (NICU). In turn, prolonged NICU stay may be associated with a higher risk of the implementation of iatrogenic procedures [31,32] and longer total postnatal hospitalization. Delivery-related stress causes the activity of various cytokines, therefore changes in this process may have impact on the developing immune system. In the Taiwanese study, Liao et al. [22] reported also that TNF-α and IL-6 response toward TLR1–2 stimulation was significantly reduced in CS delivered neonates than in those delivered vaginally (Figure 1).

The disturbed colonization with bacterial microflora within the skin and digestive tract was the reason for a more frequent occurrence of immune diseases in children delivered by cesarean section [33]. The possibility of the transfer of those bacteria to the neonatal digestive tract is higher in case of long-term contact with the vaginal flora during vaginal delivery [34]. An increasing number of authors emphasized the contribution of maternal rectal microbiome to the optimal colonization of neonates [35,36]. The colonization is also promoted by pH in the stomach of neonates, which thanks to the swallowing of the amniotic fluid in utero, becomes neutral and provides conditions for the survival of aspirated bacteria. *Lactobacillus*, *Bifidobacterium* and *Bacteroides* are the dominant species of intestinal bacteria occurring at the early postnatal period in vaginally delivered neonates [34]. The bacteria play an important part in the regulation of the immune system [37] influencing the level of NK (natural killer) cells [38], regulating the population of T lymphocytes [39,40] the secretion of IgA antibodies [41], and the synthesis of proinflammatory cytokines [42,43,44]. *Lactobacillus* bacteria may prevent airway hyperresponsiveness by limiting the presence of inflammatory cells in the peribronchial tissue [45], while *Bifidobacterium* species prevents intestinal necrosis and plays a role in the regulation of the body weight of an infant [46,47]. Numerous authors emphasized both quantitative and qualitative differences in the intestinal microflora depending on the mode of delivery completion. It was reported that the meconium of neonates delivered by cesarean section included reduced amounts of *Lactobacillus*, *Bifidobacterium*, *Bacteroides* and *Prevotella* bacteria, while the dominant ones included iatrogenic bacterial species or ones which colonized the skin, i.e., *Staphylococcus* [34], *Streptococcus* [48], *Klebsiella*, *Enterococcus* and *Clostridium* [49]. *Bifidobacterium* species, responsible for the synthesis of short-chain fatty acids (SCFA), undergo a relatively rapid elimination from the digestive tract of neonates delivered by cesarean section [34]. SCFAs are a type of communicators between the microbiome and the immune system contributing to the maintenance of balance between pro- and anti-inflammatory reactions, e.g., by transferring the signal with a group of G protein-coupled receptors (GPR), which are present not only on the cells of the gastrointestinal system, but also of the immune and nervous system. It is believed that SCFAs may modulate the weight and reduce the amount of consumed food by the stimulation of enteroendocrine L cells responsible for releasing peptide YY (PYY) and glucagon-like peptide 1 (GLP-1) [50]. Therefore, they may influence the body weight of neonates. However, the role of bacterial microflora is still ambiguous in the development of type 1 diabetes [51].

Numerous authors of epidemiological studies emphasized a correlation between environmental factors influencing the fetus prenatally and over the early postnatal period and the development of circulatory system diseases, diabetes, obesity, tumors [52,53,54,55] and schizophrenia [56] in adulthood. The Epigenetic Impact of Childbirth (EPIIC) study demonstrated that the use of oxytocin, antibiotics and cesarean section may lead to long-term health implications [57]. The authors suspected that epigenetic mechanisms which influenced gene expression modification might be responsible for the phenomenon [57]. DNA methylation is a well elucidated epigenetic mechanism. It consists in adding a methyl group to cytosine-5-carbon in a reaction catalyzed by deoxyribonucleic acid methyltransferase (DNMT). CpG island hypermethylation within a gene promoter most commonly results in the reduction or inactivation of its expression [58,59]. Research showed that tobacco smoking, malnutrition, and long-lasting maternal stress during pregnancy might lead to silencing the expression of some genes in the fetus resulting in a variety of clinical consequences [60,61,62]. According to some researchers, cesarean section changed the global DNA methylation and the methylation of individual genes. Schlinzing et al. demonstrated a higher global methylation in the leukocytes of the umbilical cord blood in a group of elective cesarean sections [63] while Słabuszewska et al. showed a significantly lower global methylation of DNA in the placenta of women following a cesarean section [64]. Franz et al. found no differences in the global methylation of DNA between vaginal deliveries and cesarean sections, although the methylation of individual genes was significantly higher in neonates delivered by cesarean section [65]. Notably, the above mentioned studies vary in terms of methodology which may contribute to differences in the results. Therefore, the issue of the influence of cesarean section on changes in DNA methylation and its clinical implications still remains the subject of research.

Therefore, we decided to review current literature concerning pediatrics consequences of cesarean section, because of a high percentage of elective cesarean sections and numerous studies providing mutually exclusive conclusions regarding possible neonatal complications following surgical deliveries.

## 2. Material and Methods

This systematic review was conducted in accordance with the Preferred Reporting Items for Systematic Reviews and Meta-Analyses (PRISMA) guideline.

### 2.1. Search Strategy

We searched PubMed, Science Direct databases for relevant papers published from 2008 to 2020 (last search August 2020). The title and abstract of the studies were screened following inclusion/exclusion criteria. Furthermore, a hand-search of the reference sections of relevant previous reviews along with reference lists of studies meeting the inclusion criteria was also conducted. The identified studies were included in accordance with the Population, Intervention, Comparison, Outcomes (PICO) model in order to select the relevant research question in the selection criteria.

- Population: newborns and children who have delivered via cesarean section
- Intervention: cesarean section
- Comparison: any mode of delivery where reported
- Outcomes: respiratory diseases, asthma, obesity, overweight, diabetes mellitus type 1, neurological disorders

The only limits applied were the date (original reports published from 2008 to 2020; review papers and meta-analyses published from 2010 to 2020) and the language (available in English).

### 2.2. Statistical Analyses

Statistical analyses were performed with the use of Review Manager software (RevMan version 5.4 Cochrane, London, UK). To determine the strength of association between the caesarean section and respiratory tract infections, asthma, diabetes mellitus type 1 as well as obesity the pooled odds ratios (OR) with the 95% confidence intervals (CI) were calculated. For caesarean section events we took into account both emergency and elective caesarean sections whereas vaginal delivery covers both unassisted and assisted vaginal delivery. To assess the degree of heterogeneity between included studies, the I^2^ is calculated. It describes the proportion of variance (from 0% to 100%), which is due to variance in true effect sizes rather than sampling error. It is assumed that in the case of significant heterogeneity between studies, the random effects method (DerSimonian–Laird) should be used to calculate the pooled OR with the 95%CI whereas in the case of nonsignificant heterogeneity, the calculation should be performed with fixed effects method (Mantel–Haenszel). Since random-effect model is supposed to provide a more conservative OR estimation than fixed model, by representing the mean association in the populations, the results of random effect model are only demonstrated in our study. To assess potential publication bias, Egger’s regression and Begg’s rank correlation were performed. In addition, to evaluate the stability of the results, sensitivity analyses were made by sequential exclusion of each study.

The obtained results of the present meta-analysis were summarized in tables as well as illustrated using forest plots.

## 3. Results

### 3.1. Study Selection Process

In total 19,175 articles were initially identified. For the initial screening, 3425 duplicates were identified and removed, leaving 15,750 articles. Titles and abstracts, were then assessed by two researchers, with this process ending with the inclusion of 1636 articles. Full texts were then retrieved for those citations considered potentially relevant and assessed for eligibility by the two researchers. Of these 1636 articles, 1479 were excluded. The most common reason for exclusion was preterm delivery, instrumental delivery, the small number of participants and language other than English. Reference lists of included studies were hand searched by the first author and a further 2 articles were subsequently included. Separate searches were performed for each of the topics covered in this review. For the first elimination step, studies that were clearly not relevant based on the title were removed. Then, duplicates were removed. Next, the remaining abstracts were reviewed and those not relevant to the topic were removed. For all the remaining papers, the full text of the paper was read to determine whether relevant information was included.

**Figure 2:**
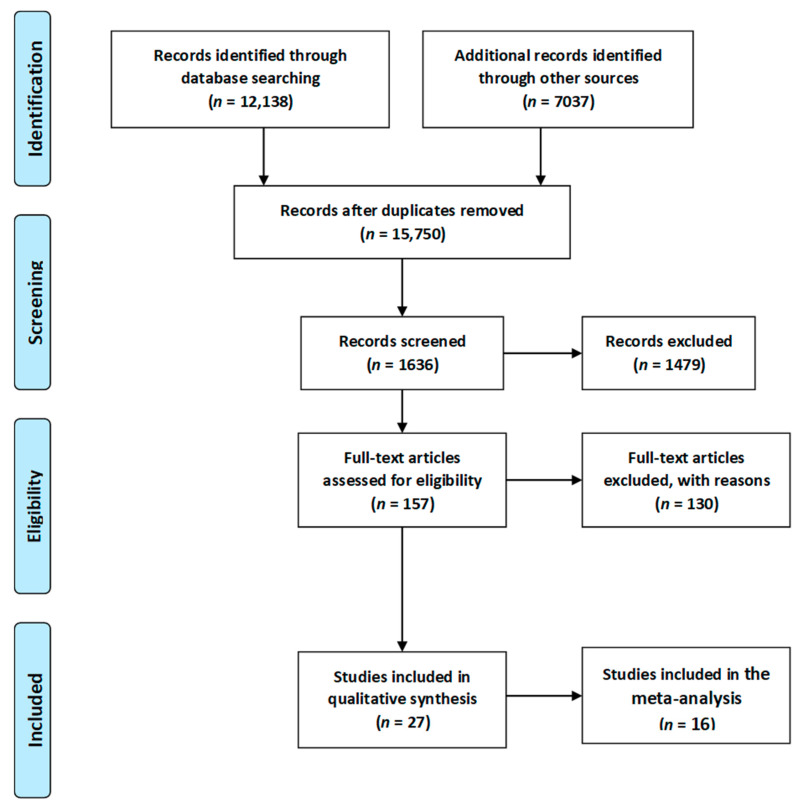
Flow chart presenting the process of searching for the eligible articles according to Preferred Reporting Items for Systematic Reviews and Meta-Analyses (PRISMA) guidelines [90].

**Figure 3:**
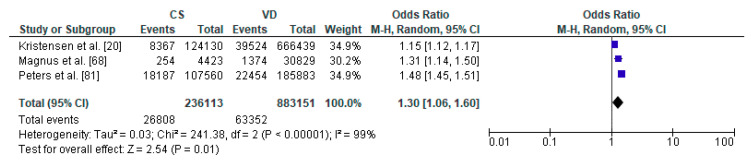
Forest plot for respiratory tract infections in the caesarean section (CS) and vaginally delivered (VD) offspring. M.-H.-Mantel-Haenszel; CI-confidence interval; I^2^-heterogeneity; df-degrees of freedom.

**Figure 4:**
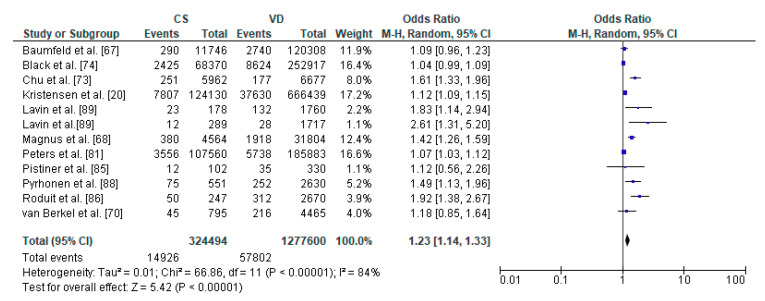
Forest plot for asthma in the caesarean section (CS) and vaginally delivered (VD) offspring. M.-H.-Mantel-Haenszel; CI-confidence interval; I^2^-heterogeneity; df-degrees of freedom.

**Figure 5:**
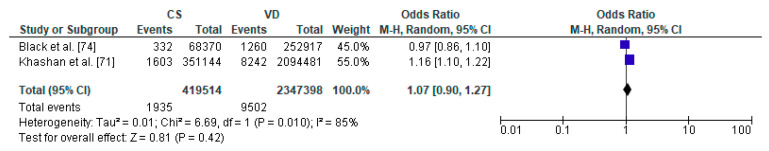
Forest plot for diabetes mellitus type 1 in the caesarean section (CS) and vaginally delivered (VD) offspring. M.-H.-Mantel-Haenszel; CI-confidence interval; I^2^-heterogeneity; df-degrees of freedom.

**Figure 6:**
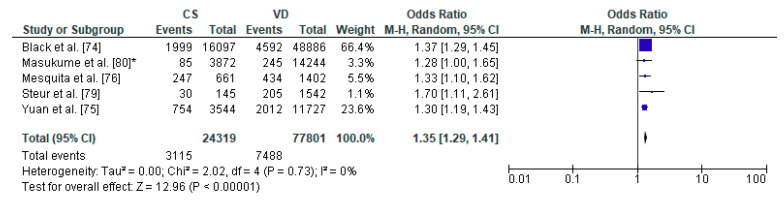
Forest plot for obesity in the caesarean section (CS) and vaginally delivered (VD) offspring. M.-H.-Mantel-Haenszel; CI-confidence interval; I^2^-heterogeneity; df-degrees of freedom; *-analysis for group of children at three years of age.

Eventually, a total of 27 relevant articles [11,20,66,67,68,69,70,71,72,73,74,75,76,77,78,79,80,81,82,83,84,85,86,87,88,89] were included in the current systematic literature review. A summary of the search process is illustrated in Figure 2, as recommended by the PRISMA guidelines [90]. Due to sufficient data regarding the occurrence of respiratory tract infections, asthma, overweight/obesity as well as diabetes mellitus type 1, 16 articles [11,20,67,68,70,71,72,73,74,75,76,77,78,79,80,81,85,86,88,89] were included in the meta-analysis. Results of the statistical tests are presented in Figure 3, Figure 4, Figure 5 and Figure 6.

### 3.2. General Characteristics of the Studies Included

Studies included in the systematic review were conducted in 5 different continents with the majority conducted in Europe (n = 18) in 15 different countries with the majority conducted in Sweden (n = 5). Twenty studies are prospective cohort while 7 are retrospective. Ten studies distinguished between types of cesarean section when reporting study results. Characteristics of studies including country, type of study design, sample size, age and assessed disorder are demonstrated in Table 1, Table 2, Table 3 and Table 4.

### 3.3. Caesarean Section and Respiratory Tract Infections—Meta-Analysis

The meta-analysis regarding the impact of caesarean section on the risk of respiratory tract infections in offspring was conducted based on 3 studies with total number of 236,113 children delivered by caesarean section and 883,151 vaginally delivered children. Children were examined within a wide range of ages, from 0 to 14 years of age (Table 1). Respiratory tract infections was significantly more common in the CS group than the VD group (pooled OR = 1.30 95%CI 1.06–1.60, *p* = 0.001) which indicates that caesarean section increased the risk of respiratory tract infections in offspring (Figure 3). No publication bias was found for this analysis [Egger’s test (*p* = 0.915); Begg’s test (Kendall’s Tau = −0.33, p = 0.601)]. The results of the meta-analysis were found stable after performing sensitivity analysis.

### 3.4. Caesarean Section and Asthma—Meta-Analysis

The relation between caesarean section and asthma in offspring was performed in meta-analysis based on 11 studies. The study by Lavin et al. [89] analyzed caesarean section within two populations, India and Vietnam which were analyzed separately. Total number of included children delivered by caesarean section were 1,791,855 and 1,277,620 vaginally delivered children. Children were examined within a wide range of age, from 0 to 18 years of age, most commonly at the age of 8 years. The authors of the included studies focused their attention on risk of asthma, asthma hospitalization and asthma symptoms (Table 2). The random effect models analysis revealed that asthma was significantly more common in the CS group than the VD group (pooled OR = 1.23 95%CI 1.14–1.33, *p* < 0.00001) which indicates that caesarean section may be a risk factor for asthma in offspring (Figure 4). The results of publication bias tests indicated that there might be potential publication bias in studies on the relationship between CS and asthma [Egger’s test (R = 2.03, *p* = 0.024); Begg’s test (Kendall’s Tau = 0.15, *p* = 0.493)]. The bias was not present when the results by Lavin et al. study [89] performed in Vietnam population were removed [Egger’s test (R = 1.94, *p* = 0.051); Begg’s test (Kendall’s Tau = 0.13, *p* = 0.586)].

### 3.5. Caesarean Section and Diabetes Mellitus Type 1—Meta-Analysis

To the meta-analysis on the relation between caesarean section and diabetes mellitus type 1 in offspring 2 studies were included with a total number of 419,514 children delivered by caesarean section and 2,347,398 vaginally delivered children. The age of children examined ranged from the first 5 years to 27 years (Table 3). The present meta-analysis showed that diabetes mellitus type 1 did not significantly differ between CS and VD groups thus caesarean section does not increase the risk factor of metabolic disorders in offspring (pooled OR = 1.07 95%CI 0.90–1.27, *p* = 0.2) (Figure 5). Due to fact that analysis was based only on two studies, both publication bias as well as sensitivity analysis were not performed and thus the results must be treated with caution.

### 3.6. Caesarean Section and Increased Body Weight—Meta-Analysis

The pooled OR for relation between caesarean section and increased body weight in offspring was analyzed for 5 studies, out of 8 articles which met the inclusion criteria of the present systematic review: a total number of 24,319 children delivered by caesarean section and 77,801 vaginally delivered children. The age of children examined ranged from 5 years to 28 years. The authors of the included studies focused their attention on overweight, obesity, risk of adiposity and body mass index and body fat (Table 4). The present meta-analysis demonstrated that increased body weight was significantly more common in the CS group than the VD group thus caesarean section may be a risk factor of obesity in offspring (pooled OR = 1.35 95%CI 1.29–1.41, *p* < 0.00001) (Figure 6). No publication bias was found for this analysis [Egger’s test (R = 0.12, *p* = 0.863); Begg’s test (Kendall’s Tau = 0.20, *p* = 0.624)] and sensitivity analysis revealed stability of the results.

## 4. Discussion

The stimulation of the hypothalamic-pituitary-adrenal axis (HPA) in the fetus leads to the increase in stress hormones contributing to lung maturation [91], thereby reducing the postnatal manifestations of respiratory insufficiency [92]. Another analysis of a multicenter World Health Organization Multicounty Survey on Maternal and Newborn Health (WHOMCS) revealed that cesarean section increased morbidity in neonates [93]. The most common complications listed by researchers include respiratory disorders, transient tachypnea or postpartum hypoglycemia [94,95]. The frequency of those complications was influenced by the co-existence of childbirth which significantly decreased the occurrence rates of such disorders [66,96]. In the case of an elective cesarean section, the risk of respiratory morbidity including transient tachypnea of the newborn (TTN), respiratory distress syndrome (RDS), and persistent pulmonary hypertension (PPH) at 37 weeks of gestation reached 10%, while with vaginal delivery the risk was 2.8% [67]. Previously, infants delivered by caesarean section were demonstrated to have significantly lower compliance of the respiratory system at the age of 1 year than those after vaginal delivery [22]. The authors however, did not observe differences in the resistance of the respiratory system and maximal expiratory flow at functional residual capacity between the groups depending on the type of delivery. Hansen et al. [66] reported that the percentage of complications was affected by cesarean section procedures and by the duration of the pregnancy. The risk of developing RDS after cesarean section at 37 gestational weeks increased 4-fold (odds ratio (OR) 3.9, 95% confidence interval 2.4 to 6.5), while at 39 weeks it was half lower (OR 1.9, 95% confidence interval (CI) 1.2 to 3.0) [66]. Similar conclusions were reached following an Israel study including 132,054 cases. Hospitalizations of offspring involving respiratory morbidity were significantly common in offspring delivered caesarean section (5.2% vs. 4.3% in vaginal deliveries [67]. Table 1 presents characteristics of studies regarding the impact of cesarean section on offspring respiratory morbidity. Currently, a prophylactic dose of corticosteroids is used in everyday clinical practice in order to avoid respiratory complications associated with elective cesarean section and premature delivery [97,98,99].

Numerous epidemiological studies demonstrated a correlation between cesarean section and an increased risk of developing immune diseases, including bronchial asthma [68,69,70,100], allergic rhinitis [101], ulcerative colitis, type 1 diabetes mellitus [17,71], celiac disease [102] and obesity [72,103]. Chu et al. [73] studied the risk of developing asthma and allergic rhinitis in case of cesarean section without medical indications. The authors reported that children delivered by cesarean section significantly more often develop asthma and allergic rhinitis [73]. Similar results were obtained in a meta-analysis conducted in 2018, which revealed a significantly higher risk of developing asthma up to the age of 12 years (OR 1.21, 13 studies, n = 887,960) and obesity up to the age of 5 years (OR 1.59, 95%CI 1.33–1.90; n = 64,113; 6 studies) in children delivered by cesarean section [23]. Comparable results concerning asthma were revealed by a meta-analysis conducted by Darabi et al. [104]. It needs to be emphasized that the above mentioned studies included the analyses of cesarean sections without considering the clinical indications for performing the procedure. Black et al. compared emergency cesarean section with elective c-section and found no significantly different risk of asthma and obesity at age 5 year, however, authors noticed increased risk of developing type 1 diabetes (0.66% vs. 0.44%; difference 0.22% [95%CI 0.13–0.31%]) [74]. A prospective study conducted in the United States included over 22,000 participants and showed a 13% cumulative risk of developing obesity in children delivered by cesarean section. The authors emphasized that the risk of developing obesity in children delivered by cesarean section increased up to 64% compared to their siblings who were delivered vaginally [75]. Mesquita et al. demonstrated that the prevalence of obesity in children delivered by cesarean section was 33% higher and in a group of 19-year olds it increased to 50% compared to their vaginally delivered peers [76]. Another European cohort study confirmed the correlation between cesarean section and the risk of developing obesity in children at the age of 3. However, the fact was only observable in a group of acute cesarean sections (adjusted relative risk ratio (aRRR) = 1.23; 95% CI 1.04–1.44), but not elective ones (aRRR = 1.06; 95% CI 0.90–1.25) [77]. Basing on the obtained results the issue of the participation of the microbiota in the development of obesity should be further discussed [51]. Ahlqvist, et al. [78] found no evidence of an association between elective or nonelective cesarean section and young adulthood obesity in young male conscripts when accounting for maternal and paternal factors (relative risk ratio RRR = 0.96; CI 95% 0.83–1.10). Authors suggested there is no clinically relevant association between cesarean section and the development of obesity. The PIAMA study indicated an increased risk of obesity (OR = 1.7) [79] while a long-term prospective study conducted in the United Kingdom showed no significant correlation between elective cesarean section and both body mass index (BMI), as well as body fat percentage (BF%) in children [80]. The results of the remaining studies concerning the risk of developing other immune diseases are also ambiguous. A European prospective study comprising over 320,000 participants showed no significant differences in the prevalence of obesity and asthma in children aged up to 5 years delivered by cesarean section regardless of the manifestations of concomitant childbirth. Moreover, the authors did not confirm significant differences regarding the frequency of obesity, intestinal inflammation, and type 1 diabetes between a group of children delivered vaginally and by elective cesarean section [74]. Another study conducted in Australia showed that children delivered by cesarean section significantly more frequently developed infections, eczemas or metabolic diseases. However, the highest risk of metabolic diseases was present in case of acute cesarean sections (OR 2.63, 95% CI 2.26–3.07) [81]. An American, prospective cohort study compered the incidence of obesity and type 2 diabetes between birth by cesarean and vaginal delivery among 33 226 women participating in the Nurses’ Health Study II, who were born between 1946–1964, with follow up through the end of the 2013–2015 follow-up cycle indicated a relative risk of obesity and type 2 diabetes among women born by cesarean (OR 1.11; 95% CI 1.03–1.19 and HR 1.46; 95% CI 1.18–1.81) [105]. Table 2, Table 3 and Table 4 shows characteristics of studies in regard to the impact of cesarean section on offspring asthma, obesity and diabetes mellitus type 1.

Cesarean section is supposed to protect the neonate and its neurological consequences may differ depending on obstetric indications. In case of an elective cesarean section performed because of breech presentation or a fetal-pelvic disproportion both the mother and the fetus are subjected to limited stress unlike with intrapartum acute cesarean section. Animal research attracted attention to behavioral disorders of the offspring of females who had undergone cesarean section [106]. However, studies concerning children delivered by cesarean section demonstrated contradictory results, which assessed the prevalence of autism spectrum disorders (ASD), ADHD or behavioral disorders [82,107], especially if the analyses comprised the effect of environmental factors. A meta-analysis of the neurological consequences of surgical deliveries published in 2019 confirmed a higher risk of developing autism spectrum disorders (OR 1.33; 95% CI, 1.25–1.41) and ADHD (OR 1.17; 95% CI, 1.07–1.26) in children delivered by cesarean section. However, findings concerning intellectual deficits, obsessive-compulsive disorders, tics and eating disorders were not so explicit. The study based on 300 children at pre-school age reported that mode of delivery has no impact on IQ score [108]. In turn, the study performed in sizeable group of 5000 pre-school children showed that children delivered by caesarean section had significantly higher IQ test scores. However, the authors observed no significant difference in IQ scores between caesarean delivery and natural vaginal delivery groups after adjusting of among others maternal and paternal education, maternal age and parity [109]. Zhang et al. [110] reported no correlation between the procedure of cesarean section and the risk of developing depression, affective and non-affective psychosis. On the other hand, Baumfeld et al. conducted a prospective cohort study and demonstrated that cesarean section was an independent factor affecting neurological disorders in children along with birth weight, maternal age, Apgar score, gestational age and the sex of the neonate [83]. Recent data by Sadowska et al. [84] demonstrated that delivery by cesarean section increased the risk of epilepsy over two-fold (OR 2.17) in the patients with cerebral palsy. Deoni et al. [111] demonstrated for the first time that caesarean section may be related with changes in brain development, at least during early infancy. The authors observed delivery mode-related differences in white matter development during infancy, which involved the frontal, temporal, and parietal lobes as well as corpus callosum. Children delivered by cesarean section showed significantly lower white matter development in widespread brain regions and simultaneously lower functional connectivity in the brain [112].

## 5. Study Limitations

A considerable amount of research concerning the issue of cesarean sections includes retrospective studies which do not comprise clinical indications for a surgical procedure [86,87,88,89] and do not refer to data regarding the duration of amniotic fluid leakage, the presence of uterine contractions and a history of fertility treatment. Furthermore, there are differences in the definitions of acute or intrapartum cesarean section. Few authors tackled the issue of the biochemical and radiological test results, which would facilitate the objectification of the postnatal status of a newborn.

## 6. Conclusions

This systematic review of literature and meta-analysis shows, that cesarean section may be associated with several pediatric complications. The results of the studies included indicated that children delivered by cesarean section more commonly developed respiratory tract infections, obesity and the manifestations of asthma than children delivered vaginally. The risk of developing diabetes mellitus type 1 or neurological disorders in offspring after caesarean section is still under discussion. Due to a high number of reciprocally exclusive study results concerning long–term pediatric sequelae it is recommended to conduct a multicenter prospective study comprising the concept of epigenetic influence of cesarean section.

## Figures and Tables

**Table 1:** Characteristics of the studies analyzing the impact of cesarean section on offspring respiratory tract infections which met the inclusion criteria of both the present systematic review and meta-analysis.

Author	Country	Study Design	Sample Size	Age	Assessed Respiratory Morbidity
Kristensen et al. [20]	Denmark	Cohort Retrospective	790,569	from 0 to 14 y	lower respiratory tract infection
Magnus et al. [68]	Norway	Cohort Prospective (Norwegian Mother and Child Cohort Study)	37,171	36 months	recurrent lower respiratory tract infections
Peters et al. [81]	Australia	Cohort Prospective	491,590	in the first 28 days and up to 5 y	respiratory infection

**Table 2:** Characteristics of studies included the impact of cesarean section on asthma in offspring, which met the inclusion criteria of both the present systematic review and meta-analysis.

Author	Country	Study Design	Sample Size	Age	Assessed Asthma Morbidity
Kristensen et al. [20]	Denmark	Cohort Retrospective	790,569	from 0 to 14 y	Risk of asthma
Baumfeld et al. [67]	Israel	Cohort Retrospective	132,054	18 y	Risk of asthma
Magnus et al. [68]	Norway	Prospective (Norwegian Mother and Child Cohort Study)	37,171	3 y	Risk of asthma
van Berkel et al. [70]	Netherlands	Cohort Prospective	6128	6 y	Risk of asthma
Chu et al. [73]	China	Case-Control Retrospective	1385	5–12 y	Risk of asthma
Black et al. [74]	UK	Cohort Retrospective	321,287	5 y	Asthma hospitalization
Peters et al. [81]	Australia	Cohort Prospective	491,590	in the first 28 days and up to 5 y	Risk of asthma
Pistiner et al. [85]	USA	Cohort Prospective	498	9 y	Asthma symptoms
Roduit et al. [86]	Netherlands	Cohort Prospective	2917	8 y	Risk of asthma
Pyrhonen et al. [88]	Finland	Cohort Retrospective	4779	4 y	Risk of asthma
Lavin et al. [89]	Vietnam	Cohort Prospective	2000	8 y	Risk of asthma
Lavin et al. [89]	India	Cohort Prospective	2026	8 y	Risk of asthma

**Table 3:** Characteristics of studies included the impact of cesarean section on diabetes mellitus type 1 in offspring which met the inclusion criteria of both the present systematic review and meta-analysis.

Author	Country	Study Design	Sample Size	Age	Assessed Metabolic Disorders
Black et al. [74]	UK	Cohort Retrospective	321,287	5 y	diabetes mellitus type 1
Khashan et al. [71]	Sweden	Cohort Prospective	2,638,083	27 y	diabetes mellitus type 1

**Table 4:** Characteristics of studies included the impact of cesarean section on problems with body weight in offspring which met the inclusion criteria of the present systematic review.

Author	Country	Study Design	Sample Size	Age	Assessed Problems with Body Weight
Black et al. [74]	UK	Cohort Retrospective	321,287	5 y	Obesity
Ajslev et al. [72]	Denmark	Cohort Prospective	28,354	7 y	Obesity
Yuan et al. [75]	USA	Cohort Prospective	22,068	20–28 y	Obesity
Mesquita et al. [76]	Brazil	Cohort Prospective	2063	23–25 y	Risk of adiposity
Masukume et al. [77]	Ireland	Cohort Prospective (GUI study)	11,134	3–5 y	Obesity
Alhqvist et al. [78]	Sweden	Cohort Prospective	97,291	9–12 y	Obesity
Steur et al. [79]	Netherlands	Cohort Prospective (PIAMA study)	1687	8 y	Overweight
Masukume et al. [80]	UK	Cohort Prospective (MCS)	18,827	14 y	Body mas index and body fat

GUI-Growing Up in Ireland; PIAMA-Prevention and Incidence of Asthma and Mite Allergy; MCS- Millennium Cohort Study.
